# Associations between neighborhood characteristics, mental health, and parenting among mothers with young children in Brazil: A cross-sectional study of women living in communities of social disadvantage and ethnic diversity

**DOI:** 10.1371/journal.pmen.0000075

**Published:** 2024-09-20

**Authors:** Clariana Vitória Ramos de Oliveira, Silvana Freire, Simoní Saraiva Bordignon, Laura Chechel, Paul Springer, Claudia Nery Teixeira Palombo

**Affiliations:** 1 School of Nursing, University of Nevada Las Vegas, Las Vegas, Nevada, United States of America; 2 New York University, New York, New York, United States of America; 3 School of Nursing, Federal University of Rio Grande, Rio Grande, Rio Grande do Sul, Brazil; 4 Virginia Tech University, Blacksburg, Virginia, United States of America; 5 School of Nursing, Federal University of Bahia, Salvador, Bahia, Brazil; University of Nevada Las Vegas, UNITED STATES OF AMERICA

## Abstract

**Background:**

Recent research has highlighted the intricate relationship between the quality of a neighborhood’s social and physical characteristics and maternal well-being in promoting healthy parenting practices and improving child development. This study examined the associations between neighborhood characteristics, maternal depressive symptoms, and parenting practices.

**Methodology/principal findings:**

A cross-sectional study in the city of Salvador, Brazil interviewed mothers of children between the ages 0 to 6 at local Primary Health Care Clinics. Neighborhood characteristics were assessed using questions adapted from the Guide for the Development of Early Childhood Friendly Neighborhoods. Maternal depressive symptoms were measured using the Self-Report Questionnaire (SRQ-20), and parenting practices were evaluated using the United Nations Children’s Fund Family Care Indicators. The study included 503 mother-child dyads. The average maternal age was 31 years, with 50% identifying as black and 45% as brown. 67.6% of households in the study earned below the minimum wage. Multivariate regression analysis adjusted for sociodemographic covariates was used to estimate the associations between maternal outcomes and four neighborhood characteristics: sense of physical and social safety, issues or problems in the community, access to public services, and access to green and child friendly spaces. Findings revealed that only perceived safety was associated with reduced maternal depressive symptoms, while access to public services in the neighborhood was inversely related to parenting stimulating practices. No other neighborhood characteristics showed a significant association with the outcomes.

**Conclusion:**

The findings revealed both expected and unexpected associations between neighborhood characteristics, maternal depressive symptoms, and parenting practices. Overall, these findings contribute to our understanding of the multifaceted dynamics between community environments and maternal well-being. They also shed important light on the various ways in which one’s environment can influence maternal mental health and parenting behaviors.

## Introduction

In recent years, the complex interplay between environment and mental health has gained significant attention within the realm of public health and social sciences. The environment in which individuals interact plays a pivotal role in shaping various facets of their lives, including mental well-being [1], and parenting practices [2–4]. With a growing emphasis on understanding these intricate relationships, researchers have turned their focus toward examining how neighborhood characteristics impact maternal and child health.

A comprehensive global systematic review, found that among low, middle and high income countries, the prevalence of maternal mental disorders/illnesses during the perinatal period were between 1.9% and 80.1% [5]. These studies also found higher prevalence rates among low-to middle-income communities, suggesting that maternal mental disorders and illnesses are associated with low socioeconomic status [5–8]. Among the Brazilian population one study found between 15%-30% of mothers experienced depression and/or anxiety, with this rate rising to an alarming 40% during the COVID-19 pandemic [9]. These data coupled with new research showed that up to 10%-13% of Brazilian women were at risk for suicide during the perinatal period, highlighting the seriousness of this dilemma [9, 10]. Because of the prevalence of maternal mental disorders and illnesses among the perinatal period, there is a growing interest in understanding how environmental factors impact the mental health and well-being of mothers.

It is important to acknowledge that the impact of neighborhood characteristics go beyond geographical borders. Rather, they encompass factors such as community safety, social cohesion, and the physical and psychosocial environments in which individuals and families navigate their daily lives. Recent studies have explored the impact that neighborhood environments have on maternal mental health [11, 12]. One study reported that green space, neighborhood communication, and neighborhood safety were all associated with better mental health and wellbeing among residents [11]. Another study found that social cohesion played an important role in how mothers perceived their neighborhood quality, with depressive symptoms higher in neighborhoods in which mothers reported lower social cohesion and higher levels of negative perceptions [12]. In the context of Brazil, which is an incredibly diverse country both in culture and socio-economic factors, additional research is needed to understand what neighborhood characteristics influence maternal mental health and parenting practices.

The documented impacts of maternal mental health on child development, is also well established [13, 14], and calls for a deeper understanding of factors that impact maternal mental health and parenting practices. Research has previously shown how neighborhood characteristics have been linked to parenting practices such as parenting style, and parent-child interactions [15]. Social environment also plays a significant role in shaping a child’s development [16]. A recent study in Brazil found that family social exclusion was associated with poor motor and socio-emotional development in children between the ages of birth to 5 [16]. Other study among Latina mothers have shown that communities marked by higher levels of violence were linked to negative parenting practices such as strict parental monitoring and physically and socially withdrawn behaviors [17].

Despite findings highlighting the impact that community environments and neighborhood factors have on maternal mental health and parenting practices, there continues to be a scarcity of research examining these characteristics among low- and middle-income countries. There is even a greater dearth of research among socially disadvantaged and ethnically diverse communities [4] like those in Brazil. To address this gap, this study examines the associations between neighborhood characteristics, maternal depressive symptoms, and parenting practices among Brazilians mothers. We hypothesize that mothers living in less favorable neighborhood conditions will exhibit higher levels of maternal depressive symptoms and lower levels of positive parenting practices.

## Methods

### Study design

This study utilized a cross-sectional design and followed the Strengthening the Reporting of Observational Studies in Epidemiology (STROBE), which are guidelines for reporting observational data. Data from the "Dimensions of Impact of the Territory on the health and nutrition conditions of children in early childhood" study, which was funded by the National Council for Scientific and Technological Development, were also utilized. This study was conducted in Salvador, Bahia, the oldest and most diverse city in Brazil. The Human Development Index (HDI) for Salvador was 0.759, placing it 89^th^ out of 193 countries or territories [18]. This statistical index compares country indicators in terms of wealth, literacy, education, life expectancy, birth rate, and others, to evaluate the well-being of a population. The population of Salvador is 2,418,005 people, with 213,765 children under six years of age [19].

### Study participants

A total of 503 dyads, including mothers and their children were enrolled in the main study. Participants were recruited through a partnership with primary care health clinics that are part of the Unified Health System of Brazil.

To participate in this study, mothers needed to be caregivers of a child under the age of 6, and reside in the city of Salvador. Participants who disclosed chronic or neurological diseases were excluded from this study. For data collection purposes, if a mother had more than one child, they were asked to answer questions related to their youngest child. Although the questions regarding maternal parenting practices were only directed toward the mothers, these questions addressed positive parenting practices and interactions these mothers had with their child between the ages 0 to 6. For the purpose of this study, the authors did not analyze specific outcomes for their child, rather only specific demographic data from these children were utilized. All participants in this study provided written informed consent.

A power analysis was run, to identify the needed sample size to run the statistical analysis. Based on this analysis, the sample size needed for this study was 420 dyads. This was based on the proportion of children with inadequate feeding practices (p = 50%), the population of children under the age of six in the 12 health districts [20], a confidence level of 95%, a margin of error of 5% and an estimation of 20% attrition. The data collected for this study well exceeded the needed sample size.

### Study data collection

Data collection occurred between January 1st to February 29^th^, of 2023, among all 12 health districts in the municipality of Salvador. The mothers and their respective children were approached at their primary care health units and invited to participate in the research study. Participants who agreed to take part of the study were interviewed by trained undergraduate research students which lasted approximately 40 minutes.

A brief sociodemographic questionnaire was used to record sociodemographic variables. This included variable such as age, race, gender, educational level, marital status, religion, working status, and monthly family income. Assessment instruments were given to all participants to access perceptions of their neighborhood environment, their own maternal mental health, as well as their parenting practices.

### Ethical aspects

This study obtained approval from the ethics committee of a Federal University in Northeast Brazil. All participants signed the Free and Informed Consent Form, and the current norms for Research on Human Beings were followed. This included following the National Research Ethics Commission, Resolution 466/2012, related to human subjects research that ensures the autonomy, justice, equity, beneficence and non-maleficence of each of the research participants.

### Exposure outcomes variables

#### Neighborhood characteristics

The neighborhood characteristics were evaluated based on questions adapted from the Guide for the Development of Early Childhood Friendly Neighborhoods [21]. These questions were developed by the Institute of Architects of Brazil to assist policymakers in considering what the needs of infants, young children, and their caregivers are, and how these needs should be taken into account when developing safe, green, accessible and inclusive neighborhoods.

To reduce data dimensionality, twelve questions related to neighborhood characteristics were analyzed using a principal axis factor analysis with Promax (oblique) rotation (Table 1). The analysis yielded four meaningful factors explaining a total of 141% of the total variance. Factor 1 was labeled as *“Sense of physical and social safety in the community”* and included three items: safe access to recreational areas, sense of safety, and perceptions of social cohesion in the neighborhood with factor loadings ranging from .432 to .636. Factor 2 was labeled as *“Issues or problems in the community”* and included three items: the noise level, flooding, and trash in the neighborhood with factor loadings ranging from .488 to .587. Factor 3 was labeled *“Access to public services”* and included three items: adequate bus stops, lighting, and other public services with factor loadings ranging from .420 to .542. Finally, Factor 4 was labeled *“Access to green and child friendly spaces”* and included three items: access to public spaces with trees, streets with stroller ramps, and play spaces for children with factor loadings ranging from .351 to .483. With the oblique rotation solution, the proportion of variance explained by each of the four factors was 0.63, 0.52, 0.49, and 0.40 respectively. Based on these results four additive indexes were generated, ranging from 0 to 3. The four indices based on the four identified factors included the following items:

**Table 1:** Neighborhood characteristic factors were analyzed using an exploratory factor analysis with Promax (oblique) rotation.

Items	Factor loadings			
	Factor 1	Factor 2	Factor 3	Factor 4
Are recreational areas in your neighborhood safely located and easily accessible?	0.602			
Do you feel safe walking or cycling around your neighborhood?	0.636			
In your neighborhood do you feel safe because your neighbors, relatives and acquaintances form a trusted community network and are generally involved and attentive to the child?	0.432			
Does your neighborhood have a lot of noise?		0.512		
Is it common to have floods in your neighborhood?		0.488		
In your neighborhood, do you see a lot of garbage in the street/open sewer?		0.587		
Are there adequate and well-located bus stops in your neighborhood?			0.420	
Do the streets in your neighborhood have adequate lighting?			0.465	
In your neighborhood, do you have easy access to services, such as commerce, schools, day care, health centers, social centers, others?			0.541	
Do you think your neighborhood has play spaces or active and attractive facades for children?				0.468
Do the sidewalks in your neighborhood have stroller ramps?				0.351
Are the streets in your neighborhood tree-lined?				0.483

Two items were dropped during the factor analysis stage (presence of trees in the neighborhood, and closing of streets for public events) because these didn’t correlate with the identified four factors.

### Outcome variables

#### Maternal depressive symptoms

Maternal depressive symptoms were assessed utilizing the Self-Report Questionnaire (SRQ-20). The SRQ-20 is a screening tool for common mental disorders, developed by the World Health Organization [22]. The SRQ-20 is made up of twenty Yes/No questions, to assist in identifying a range of symptoms including sadness, lack of energy, sleep disturbances, thoughts of self-harm, nervousness, tension, panic attacks, headaches, poor digestion, and bodily pains.

The SRQ-20 is widely used in low-and-middle-income countries, due to its practicality and objectivity [23–25], and has been shown to be a valid measure among the Brazilian population [26]. In addition, the SRQ-20 has been administered with great success in primary care settings in Brazil as well as among low- and middle-income countries [24, 26]. The instrument’s cutoff scores based on Brazilian studies range between seven and nine [27]. A binary indicator was created using the cut-off of ≥ 9 to indicate mothers who exhibit moderate-to-severe depressive symptoms.

#### Parenting practices

Parenting practices were reported as the number of positive stimulation practices a mother performed with their child. These practices were adapted from the United Nations Children’s Fund Family Care Indicators [28] and consists of 6 play and learning activities a mother engaged in with their child in the past three days. These include reading books or looking at pictures; telling stories; taking the child outside of the home; playing with the child; naming, counting, or drawing things with the child; and talking with the child while doing working or homework. These six questions are widely used in the research and have been shown to be a reliable, and valid measures with predictive validity for early child development outcomes [29–31].

A total stimulation score was achieved by summing the number of activities that a mother engaged in with their child (ranging from 0–6). Higher values indicated greater maternal stimulation practices and lower values indicated lower maternal stimulation practices. For the purpose of this study, two categories were created: (1) low maternal stimulation with scores ranging from 0 to 3 items and (2) high stimulation practices with scores ranging from 4–6.

### Data analysis and processing

Descriptive statistics were first analyzed by calculating the frequencies, means, and standard deviations for all study variables. Next, we examined the unadjusted and adjusted associations between the four neighborhood indices, maternal depressive symptoms and maternal stimulation practices. Ordinary least squares linear regression models were estimated for the continuous outcomes. Normality of the two outcome variables was assessed using the Shapiro-Wilk test. The test indicated that the outcomes deviated from a normal distribution (p < .05). As a robustness check, logistic regressions were estimated for the binary version of the depressive symptoms and parenting practices outcomes.

All the estimated models included fixed effects for the 12 Health Districts in Salvador, Brazil. The adjusted models controlled for sociodemographic variables including maternal age and categorical variables for mother’s race, education (whether mother completed secondary school or more), marital status, religion, and employment. The model also controlled for categorical variables of household characteristics such as living in a house vs. an apartment, the number of adults and children living in the home, whether the mother participated in Brazil’s cash transfer programs, and whether the household income was below the minimum wage. For categorical covariates with missing values (see Table 1), models include a binary indicator for the “don’t know/missing” category. Mother’s reports of household income had 5 missing values (< 1% of data), which were mean imputed before generating the indicator for household below minimum wage. For the linear regression models, continuous variables were standardized to have a mean of 0 and a standard deviation of 1 by subtracting its mean and diving by its standard deviation. Standardized coefficients (β) were reported for in the results section. For logistic models, Odds Ratios (OR) were reported. All the analyses were conducted using Stata 18.

### Quality control

Quality control measures included the use of pre-tested and standardized instruments; the preparation of a manual with detailed guidelines for conducting the interviews and training for the team responsible for data collection. In addition, a random sample of 5% of the interviews were reviewed by one of the lead researchers to verify the accuracy, credibility, quality and veracity of the data.

## Results

The presented results are from 503 dyads. Maternal and household characteristics are presented in Table 2. On average, mothers in this sample were 31 years of age (SD = 7.30). Approximately 50% of the mothers identified as black and about 45% identified as brown. The majority of the sample, identified as Catholic or Evangelical (60%). Approximately, 57% of mothers had completed 12 or more years of schooling, which is equivalent to completing High School in the U.S. Only 23 mothers (5%) reported being in a relationship with the child’s father. Most participants (62%) were not employed at the time of the survey, with 22% working as self-employed and only 15% reporting having a formal job. Three-quarters of the participants reported living in a house (75%), while the remaining 25% reported living in apartments. The majority of participants (68%) reported earning an income below minimum wage ($267 per month). Finally, over 65% of mothers reported participating in the cash transfer program (Bolsa Família), which is a government program aimed at alleviating poverty.

**Table 2:** Maternal household characteristics reported by study participants in Salvador, Bahia, Brazil, 2023 (n = 503).

*Maternal characteristics*	
Maternal age (years), *mean (SD)*	30.88 (7.30)
Maternal race, *n(%)*	
Black	250 (49.7%)
Brown	224 (44.5%)
Other^a^	29 (5.8%)
Maternal secondary education, *n(%)*	
Completion of 12 years or more of schooling	286 (56.9%)
Less than 12 years of schooling	217 (43.1%)
Maternal marital status, *n(%)*	
Partnered, but not with father of child	297 (59.1%)
Partnered with father of child	23 (4.6%)
Don’t know/Missing	183 (36.4%)
Maternal religion is Catholic/Evangelical, *n(%)*	
Yes	301 (59.8%)
No^b^	202 (40.2%)
Maternal employment, *n(%)*	
Employed	76 (15.1%)
Self-employed	109 (21.7%)
Unemployed	312 (62%)
Missing	6 (1.2%)
Maternal depressive symptoms, *mean (SD)*	5.66 (4.44)
Risk for maternal depression (SRQ-20 > = 9), *n (%)*	
Yes (SRQ-20 > = 9), *n (%)*	116 (23.1%)
No (SRQ-20 < 9), *n (%)*	387 (76.9%)
Maternal stimulation practices, *mean (SD)*	2.47 (1.88)
Maternal stimulation practices (categorical), *n (%)*	
High stimulation practices (> = 4)	158 (31.4%)
Low stimulation practices (< 4)	345 (68.6%)
Perceptions of community environment, *mean (SD)*	
Sense of physical and social safety	1.42 (1.13)
Issues or problems in the community	1.63 (1.06)
Access to public services	2.37 (0.88)
Access to green and child friendly spaces	0.94 (0.95)
***Household characteristics***	
Housing type, *n (%)*	
House	378 (75.2%)
Apartment	125 (24.9%)
Household income is below the minimum wage (R$ 1320), *n (%)*	
Yes	340 (67.6%)
No	163 (32.4%)
Total children/adolescents in household, *mean (SD)*	1.80 (0.96)
At least one child in the household, *n (%)*	228 (45.3%)
Two children/adolescents in the household, *n (%)*	186 (37%)
Three or more children/adolescents in the household, *n (%)*	89 (17.7%)
Total adults living in household, *mean (SD)*	2.04 (1.25)
One adult in the household, *n (%)*	122 (24.3%)
Two adults in the household, *n (%)*	303 (60.2%)
Three adults in the household, *n (%)*	47 (9.3%)
Four or more adults in the household, *n (%)*	31 (6.2%)
Household participates in cash transfer program, *n (%)*	
Yes	329 (65.4%)
No	174 (34.6%)

Notes. ^a^Other includes White (4.4%), Indigenous (1.2%) and Yellow (0.2%).

^b^ “No” includes participants who reported Camdomblé/Umbanda religion (2.6%), Espírita (0.4%), other religions (3.6%) or did not have a religion or refused to answer (33.6%).

Mothers in this sample reported moderate levels of psychosocial distress (see Table 2). The mean score for maternal depressive symptoms was 5.66 out of a maximum possible score of 20 (SD = 4.44). Despite this mean score, nearly a quarter of the sample (23%) reported a score of 9 or more, which places these mothers at-risk for depression.

Descriptive results from the maternal parenting practices index showed that mothers in the sample reported engaging in an average of 2.47 stimulation practices out of a maximum score of 6 (SD = 1.88). In other words, 68% of the mothers engaged in low maternal stimulation practices while nearly a third of mothers (31%) reported engaging in high stimulation practices with their child (see Table 2).

In terms of neighborhood characteristics, mothers in this sample reported feeling low levels of perceived access to green spaces as well as child friendly spaces, with a mean score of 0.94 out of a maximum possible score of 3 (SD = 0.95). On the other hand, mothers reported having greater access to public services with a mean score of 2.37 out of a maximum possible score of 3 (SD = 0.88). Next, mothers reported their sense of physical and social safety in their community was slightly below the index’s midpoint with a mean score of 1.42 (SD = 1.13). They also reported their perception of problems in the neighborhood that they live was slightly above the index’s midpoint with a mean score of 1.63 (SD = 1.06). These results suggest that, on average, mothers reported feeling somewhat unsafe in their neighborhood, as higher scores, indicating greater safety, were closer to 3. The reported scores were below the mean of 1.5, reflecting a perception of lower safety. In addition, they were unsatisfied with the level of noise and trash in their neighborhoods.

### Neighborhood factors and maternal mental health

Table 3 summarizes the unadjusted and adjusted associations between neighborhood factors and maternal depressive symptoms. In both the unadjusted and adjusted models, the mother’s sense of physical and social safety was significantly negatively associated with maternal depressive symptoms (Unadjusted: β = -0.20; 95% CI: -0.30, -.11; p<0.001; Adjusted: β = -0.17; 95% CI: -0.28, -0.07; p<0.01). This pattern was consistent when maternal depressive symptoms were measured as a continuous and as a binary indicator (SRQ-20 > = 9) (Unadjusted: OR = 0.68; 95% CI: 0.54, 0.86; p<0.001; Adjusted: OR = 0.71, 95% CI: 0.54, 0.93; p<0.05). This suggests that a mother’s sense of physical and social safety plays a significant role in the presence of depressive symptoms. None of the other neighborhood factors appeared to be significantly associated with maternal depressive symptoms.

**Table 3:** Unadjusted and adjusted associations of neighborhood factors with maternal depressive symptoms (n = 503). Salvador, Bahia, Brazil, 2023.

Predictors	Unadjusted results		Adjusted results^1^	
	SRQ20	SRQ20 > = 9	SRQ20	SRQ20 > = 9
	*β (95% CI)*	*OR (95% CI)*	*β (95% CI)*	*OR (95% CI)*
Sense of physical and social safety	-0.201***	0.68***	-0.174**	0.71*
	(-0.30, -.11)	(0.54, 0.86)	(-0.28, -0.07)	(0.54, 0.93)
Issues or problems in the community	0.080	1.20	0.054	1.13
	(-0.02, 0.18)	(0.95, 1.53)	(-0.04, 0.15)	(0.87, 1.48)
Access to public services	-0.055	0.94	-0.035	1.02
	(-0.15, 0.04)	(0.71, 1.24)	(-0.13, 0.06)	(0.75, 1.38)
Access to green and child friendly spaces	-0.001	0.90	0.064	1.01
	(-0.09, 0.09)	(0.70, 1.16)	(-0.03, 0.16)	(0.75, 1.36)

^1^Adjusted for mother age, mother race, mother education, mother marital status, mother religion, mother employment status, type of housing, number of adults in household, number of children/adolescents in household, access to cash transfer, access to health plan, household income below minimum wage, and the neighborhood factors. Models included fixed effects by Health District. Mean VIF for adjusted models = 1.95. Coefficients from linear models are standardized β with 95% confidence intervals in parentheses. Odds Ratios (OR) from logistic models are presented for binary outcomes with 95% confidence intervals in parentheses.

* p<0.05

** p<0.01

*** p<0.001.

### Neighborhood characteristic factors and parenting practices

Table 4 summarizes the unadjusted and adjusted associations between neighborhood factors and maternal parenting practices. In both the unadjusted and adjusted models, mother’s reporting having higher access to public services were negatively associated with the number of maternal stimulation practices they engaged in (Unadjusted: β = -0.10; 95% CI: -0.20, -0.01; p<0.05; Adjusted: β = -0.14; 95% CI: -0.23, -0.04; p<0.01). This was also the case when assessing the association between access to public services and whether mothers engaged in at least four stimulation activities with their child (Unadjusted: OR = 0.75; 95% CI: 0.57, 0.97; p<0.05; Adjusted: OR = 0.68; 95% CI: 0.51, 0.90; p<0.01). While the mother’s perception of issues and problems in the community was negatively associated with maternal parenting practices in the unadjusted model (β = -0.11; 95% CI: -0.21, -0.01; p<0.01), after adjusting for potential confounding variables (i.e., maternal and household characteristics such as education level, marital status, employment status, household income, number of adults in household, access to cash transfer program and health plan), this association was no longer statistically significant (β = -0.08; 95% CI: -0.19, 0.02; p = 0.12).

**Table 4:** Unadjusted and adjusted associations of neighborhood factors with parenting practices (n = 503). Salvador, Bahia, Brazil, 2023.

Predictors	Unadjusted results		Adjusted results^1^	
	Stimulation Practices	Stimulation Practices > = 4	Stimulation Practices	Stimulation Practices > = 4
	*β (95% CI)*	*OR (95% CI)*	*β (95% CI)*	*OR (95% CI)*
Sense of physical and social safety	0.006	1.02	0.014	1.01
	(-0.09, 0.11)	(0.83, 1.25)	(-0.10, 0.13)	(0.79, 1.28)
Issues or problems in the community	-0.109*	0.77	-0.083	0.80
	(-0.21, -0.01)	(0.62, 0.96)	(-0.19, 0.02)	(0.63, 1.03)
Access to public services	-0.104*	0.75	-0.135**	0.68
	(-0.20, -0.01)	(0.57, 0.97)	(-0.23, -0.04)	(0.51, 0.90)
Access to green and child friendly spaces	0.077	1.22	0.069	1.26
	(-0.02, 0.17)	(0.98, 1.53)	(-0.03, 0.17)	(0.97, 1.63)

^1^Adjusted for mother age, mother race, mother education, mother marital status, mother religion, mother employment status, type of housing, number of adults in household, number of children/adolescents in household, access to cash transfer, access to health plan, household income below minimum wage, and the neighborhood factors. Models included fixed effects by Health District. Mean VIF for adjusted models = 1.95. Coefficients from linear models are standardized β with 95% confidence intervals in parentheses. Odds Ratios (OR) from logistic models are presented for binary outcomes with 95% confidence intervals in parentheses.

* p<0.05

** p<0.01

*** p<0.001.

## Discussion

The results of this study shed important light on the complex interplay between neighborhood perceived safety, maternal mental health and parenting practices among a vulnerable population in Brazil (e.g., racially, ethnically and socially). A total of 503 mother-child dyads agreed to participate in this study through their access to their primary care health clinics in the northeastern part of Brazil. Results of the study not only highlighted the degree to which mothers reported moderate levels of psychosocial distress and low-stimulation parenting practices with their children, but the degree to which perceived safety in their neighborhoods was strongly associated with reduced maternal depressive symptoms. In addition, it was revealed that increased access to public services was inversely associated with mothers providing stimulation activities to their children. This is a result that merits further research and discussion.

In line with our hypothesis, mothers who reporting living in neighborhoods they perceived as safe resulted in them reporting fewer depressive symptoms compared to mothers who perceived their neighborhoods as unsafe. This result is aligned with other research studies that have shown that positive perceived safety was associated with enhanced mental well-being among adults [11] and that neighborhood perceptions among mothers improved maternal and child mental health and well-being [32]. Additional studies have found support for neighborhood environmental factors such as access to green space [33], neighborhood communication and density of fitness facilities [11] as having a significant impact on maternal mental health. These results highlight the substantial impact that the environment has on maternal mental health.

Other studies have shown the impact that environmental factors have on the maternal endocrine pathways such as stress hormones (e.g., cortisol) [34], as well as the influence biomedical factors on maternal physical health, including risks of cardiovascular disease, obesity, sleep disorders and mental health disorders [35–38]. Despite these findings solely addressing biomedical factors falls short of promoting maternal mental health [34]. An integrative and holistic approach to improving neighborhood characteristics is urgently needed to enhance maternal health practices, especially among vulnerable and marginalized populations. This studies results, have important implications for policy and community outreach programs, suggesting that interventions should be aimed at improving neighborhood safety, as a means to decrease maternal depression rates. Public health strategies must address societal and structural issues that affect neighborhood safety, green space and access to quality social programs especially among marginalized and underserved communities.

In addition to these findings, our study highlighted the inverse association between reported access to public services and maternal stimulation practices. Contrary to our initial hypothesis, this result indicated that mothers with greater access to essential public services such as adequate bus stops, and access to schools, tended to be less involved in stimulation/parenting practices with their children. While this finding was contrary to our initial hypotheses, it raises important questions about the quality and availability of these services. For example, one study found that mothers among marginalized communities reported not trusting the services that were available to them [39]. This could result in a greater reluctance to utilize the services, and a disbelief that these services could meet their needs. While other studies have shown that service accessibility can reduce negative parent-child interactions, such as maltreatment and neglect, increasing awareness of these services may have a more immediate preventive effect [39]. It is also important to recognize that awareness of or access to community services does not necessarily lead to more opportunities for parents to interact with their children. It would, therefore, be important to explore what other approaches can improve parent-child interactions, especially among vulnerable communities.

It is clear from this study, and others, that low and middle-income countries continue to face challenges in providing adequate maternal stimulation practices that can lead to improved child outcomes [40–42]. Further research is essential to identify contextual factors that may hinder or facilitate caregivers’ involvement in stimulating activities with children [40]. Also, additional studies are warranted to investigate hidden factors related to public service access and the promotion of maternal stimulation practices.

Finally, while this study failed to highlight risks factors associated with mother’s low levels of perceived access to green space, child-friendly spaces and higher levels of community noise with maternal mental health; other studies have found these variables as significant risk factors [43, 44]. For example, a study in Denmark found that children are at a higher risk of developing psychiatric disorders if they grow up in environments with few green spaces [43]. Another study highlighted the negative association between neighborhood chaos and child executive function [44]. Additional research is necessary to examine which aspects of the environment are essential to ensure that mothers can provide nurturing care to their children.

### Limitations

This study has several limitations that warrant consideration for future research. First, is the cross-sectional nature of the study. While the findings provide compelling and important evidence related to neighborhood characteristics, mental health, and parenting practices among Brazilian mothers, it is not possible to determine the direction of causality between these variables. Second, all participants were recruited from public primary health facilities resulting in a convenience sample. Since the sample was not selected through random selection, the results cannot be generalized to the larger population of Brazil. Despite this, our study had a robust sample size and sufficient power to run our analyses. Third, the data were dependent on the mother’s self-report, which can be affected by several challenges including social desirability, recall bias, and question interpretation. To combat these challenges the research team was rigorously trained in data collection procedure, and in working with participants in answering questions they may have with the self-reported measures. Despite this, self-report measures are a highly utilized and common sampling procedure. Finally, while the parental practice measures used are valid and reliable, they are relatively brief and may not capture the duration and quality of parental stimulation practices. Notwithstanding these limitations, our study contributes important findings that not only advance knowledge regarding the impact of neighborhood conditions, maternal mental health, and parenting practices but also provide policy and outreach implications to the field.

## Conclusion

In conclusion, the results of this study’s emphasize the importance that neighborhood characteristics such as perceived neighborhood safety, and access to public services has in maternal well-being and parenting practices. Policymakers and healthcare professionals must recognize the multifaceted determinants of maternal depressive symptoms and maternal-child interactions to develop targeted interventions that promote the health and development of mothers and children, particularly within underserved communities.
